# Theta oscillations: A rhythm difference comparison between major depressive disorder and anxiety disorder

**DOI:** 10.3389/fpsyt.2022.827536

**Published:** 2022-08-03

**Authors:** Yu Zhang, Lei Lei, Ziwei Liu, Mingxue Gao, Zhifen Liu, Ning Sun, Chunxia Yang, Aixia Zhang, Yikun Wang, Kerang Zhang

**Affiliations:** ^1^Department of Psychiatry, First Hospital of Shanxi Medical University, Taiyuan, China; ^2^Laboratory of Artificial Intelligence Assisted Diagnosis and Treatment for Mental Disorder, First Hospital of Shanxi Medical University, Taiyuan, China; ^3^Department of Mental Health, Shanxi Medical University, Taiyuan, China

**Keywords:** major depressive disorder, anxiety disorder, EEG power spectrum, diagnosis, biomarker

## Abstract

**Background:**

Due to substantial comorbidities of major depressive disorder (MDD) and anxiety disorder (AN), these two disorders must be distinguished. Accurate identification and diagnosis facilitate effective and prompt treatment. EEG biomarkers are a potential research hotspot for neuropsychiatric diseases. The purpose of this study was to investigate the differences in EEG power spectrum at theta oscillations between patients with MDD and patients with AN.

**Methods:**

Spectral analysis was used to study 66 patients with MDD and 43 patients with AN. Participants wore 16-lead EEG caps to measure resting EEG signals. The EEG power spectrum was measured using the fast Fourier transform. Independent samples *t*-test was used to analyze the EEG power values of the two groups, and *p* < 0.05 was statistically significant.

**Results:**

EEG power spectrum of the MDD group significantly differed from the AN group in the theta oscillation on 4–7 Hz at eight electrode points at F3, O2, T3, P3, P4, FP1, FP2, and F8.

**Conclusion:**

Participants with anxiety demonstrated reduced power in the prefrontal cortex, left temporal lobe, and right occipital regions. Confirmed by further studies, theta oscillations could be another biomarker that distinguishes MDD from AN.

## Introduction

As two major mental illnesses in society, major depressive disorder (MDD) and anxiety disorder (AN) are normally expressed as “low mood”, “unhappy”, “nervous”, or “anxious”. Both diseases are widely prevalent in the general population and primary health care (1, 2) and are difficult to distinguish (3). According to studies, 39% of people with a generalized anxiety disorder (GAD) also met the criteria for MDD (4). About 85% of people with MDD can also have significant anxiety symptoms, and up to 90% of people with AN may acquire depression as a co-disease (5). Therefore, it is critical to distinguish AN from MDD, as the two require distinct interventions. However, no definite diagnostic distinction exists between the two diseases (6, 7). The indicators of related anxiety and depression scale are subjective and imprecise. Therefore, there is an urgent need to discriminate between two groups using alternative methods. MDD and AN are two neuropsychiatric disorders (8, 9) that require a positive diagnosis to facilitate proper treatment.

EEG biomarkers have become an emerging topic in the neuropsychiatric field (10). EEG development exhibits potential desirable application in the medical diagnosis of MDD (11). In 2020, Trambaiolli et al. (12) demonstrated that by incorporating neurophysiological biomarkers, EEG can be used to predict MDD and AN as measured by widely used questionnaires. EEG biomarkers are objective indicators of a patient's medical state that are sensitive and specific to a given pathology (13). The time-series changes in resting-state EEG signals were relatively simple, and the changes in time-varying signals in the stimulus or response-locking trials were negligible. Therefore, it is advisable to use the method of spectrum analysis to decompose the complex time history waveform into several single harmonics through Fourier transform to obtain the frequency structure of the signal and the information of each harmonic and phase (14).

Dell'Acqua et al. (15) indicated that increased theta frequency in patients with depression may be associated with positive symptoms such as restlessness. Shabah et al. (16) demonstrated in multiple rat experiments that hippocampal theta modulates the behavioral inhibitory system that controls anxiety without false positives (even with sedatives) or negatives (even with drugs not effective for panic or depression), and in subsequent volunteer trials reached the same conclusion. Right frontal theta rhythm is positively correlated with neuroticism and trait anxiety and may serve as a biomarker for anxiety disorders. Frontal midline theta (FM-θ) which has already been suggested as a potential marker of anxiety, may help distinguish anxiety symptoms in pleasant or unpleasant tasks and is enhanced after anxiety symptoms are relieved (17). At the same time, previous research also contemplates whether FM-θ in resting EEG can be used as a biomarker to distinguish between MDD and AN. Therefore, this study explores this inference with great interest.

To sum up, EEG can be used to distinguish between MDD and AN. In that context, theta oscillations may be a potential discriminating indicator, but it has not been thoroughly studied at present. Therefore, this study aims to explore the differences in the theta oscillations EEG power spectrum at 4–7 Hz by collecting resting-state EEG of the two diseases.

## Materials and methods

### Subjects

The participants for this study included eligible 109 outpatients and inpatients, including 66 patients with MDD and 43 patients with AN. The patients were informed about the routine examination required before enrollment and any issues that required care. They were asked to sign an informed consent before enrollment. All patients included in the study met the diagnostic criteria of the Diagnostic and Statistical Manual of Mental Disorders, Fifth Edition (DSM-V) (18). MDD is diagnosed according to the “Depressive Disorders” chapter, requiring patients to meet the criteria for single-episode major depressive disorder. Enrolled patients with anxiety disorders met the DSM-V diagnostic criteria for unspecified anxiety disorder. According to DSM-V, it must be ensured that patients are healthy, and have no bad habits (no long-term history of smoking and drinking). Exclusion criteria included neurological trauma, neurocognitive impairment, taking any psychotropic drugs (19), participation in clinical trials within 3 months, pregnancy or contraindications, taking central stimulants, and unable to cooperate with the completion of EEG collection.

All participants completed a demographic questionnaire. Two trained evaluators independently scored patients according to the 17-item Hamilton Depression Scale (HAMD-17) (20) and the 14-item Hamilton Anxiety Scale (HAMA-14) (21). They ensured that the HAMD-17 scores in the depression group were greater than 17 points and the HAMA-14 scores were not higher than 7 points. Participants with at least 2 weeks of depressed mood, sleep disturbance, poor appetite, and even self-harm thoughts or behaviors were assigned to one group. In the anxiety group, the HAMA-14 score of the anxiety group was greater than 14 points twice, and the HAMD-17 score was not higher than 7 points. Participants who scored at least 14 points and had psychentonia, somatotonia, and autonomic nerve dysfunction symptoms for at least several months were assigned to the AN group.

Demographics are listed in Table 1. The age of the MDD group ranged between 20 and 60, while in the AN group it ranged between 18 and 65. Participants who completed the study's eligibility requirements and volunteered to participate in the clinical study were included.

**Table 1:** Demographic characteristics and scale scores of patients.

	**MDD (*n* = 66)**	**AN (*n* = 43)**	**X^2^/t**	***p*-value**
Gender (M/F)	18/48	14/29	0.588	0.256^a^
Age (years)	40.67 ± 1.62	49 ± 1.80	−3.356	0.154^b^
Education	4.48 ± 0.18	3.84 ± 0.21	2.313	0.151^b^
HAMD-17	16.45 ± 0.58	8.70 ± 0.57	9.115	0.209^b^
HAMA-14	8.02 ± 0.47	19.23 ± 1.00	−11.301	0.000^b^^*^

a p-value for chi-square test.

b p-value for double sample t-test.

* p < 0.05.

Age-related differences were not very significant between the two groups (*F* = 2.062, *P* = 0.154). The education distribution between MDD group and AN group suggested no large difference (*F* = 2.092, *P* = 0.151). The gender disparity between two groups was not significant (*F* = 1.303, *P* = 0.256). As we did not observe any significant differences in age, gender, and education between two groups, the influence of demographics to the groups difference was negligible. The average score of HAMD-17 was basically identical (*F* = 0.159, *P* = 0.209). Research reveals that HAMD-17 score in the normal population is high. Many people with AN may acquire depression symptom as a co-disease. The average HAMD-17 score of the MDD group is higher than that of the AN group. In a state of HAMA scale, scores have significant differences between MDD group and AN group (*F* = 15.79, *P* = 0.000). The average HAMA-14 score of AN group was obviously higher than that of MDD group.

### Resting-state EEG collecting

Before the experiment, participants were instructed to ensure they had enough sleep the night before their date and to avoid alcohol, caffeine, nicotine, and neuro stimulants. Upon arrival, they first signed the informed consent and were then administered the HAMA score. To maintain the study's data quality, participants were made to sit in a quiet, confined, non-direct light source stimulation environment. After sensors were installed, EEG was recorded over a 5-min period for each participant. During the experiment, participants were required to remain still with eyes open, minimizing significant facial and eye movements. Using an electrical cap at 16 scalp locations, electroencephalograms (EEG) of participants were recorded. Sixteen lateral electrode pairs (FP1, FP2, F3, F4, F7, F8, C3, C4, P3, P4, T3, T4, T5, T6, O1, and O2) were recorded, with the mastoid electrode (A1-A2) serving as the reference electrode.

### Data preprocessing

The sample rate was 256 Hz, with pass filtering of 0.1–70 Hz. Additionally, ECG records were used to investigate heart rate variability for eliminating ECG interference. The data were averaged for each electrode recording period, and the absolute power was calculated for each band. Data were segmented into three second segments. The electroencephalogram was manually corrected using independent component analysis (ICA) to remove components associated with artifacts such as blinking, eye movements, and sport-related artifacts (22–26). The investigation excluded frequencies other than 0.1–70 Hz on any channel.

### Frequency-domain analysis

The fast Fourier transform (FFT) (27) was used to transform these EEG signals from the time domain to the frequency domain, and the obtained frequency domain sampling sequence was properly converted and processed by EEGLAB to obtain the frequency spectrum of the signal, which was convenient to analyze the characteristics of the signal. A comparative study was undertaken to determine the differences in EEG power at each electrode site in theta oscillations (4–7 Hz) in anxiety and depression patients. In the frequency domain, linear time-invariant systems were generally applicable. The time variable in the resting state was negligible, indicating that we could calculate the energy difference between the two using frequency domain analysis. The complex data preprocessing steps were implemented in EEGLAB (28, 29).

### Statistical analysis

The clinical data were statistically analyzed using SPSS 26.0. The measurement data were expressed in the form of “mean ± standard deviation (x ± s)”, and the results were expressed as percentages (%). The chi-square test was used to compare the gender component in the two groups, and the two-sample *t*-test was used to compare the age, education level, and anxiety and depression scales of the two groups. EEG data were evaluated by independent samples *t*-test to evaluate the differences between groups in Weighted Phase-Lag Index (wPLI) (30), *p* < 0.05 indicated statistical significance.

## Results

**Figure 1:**
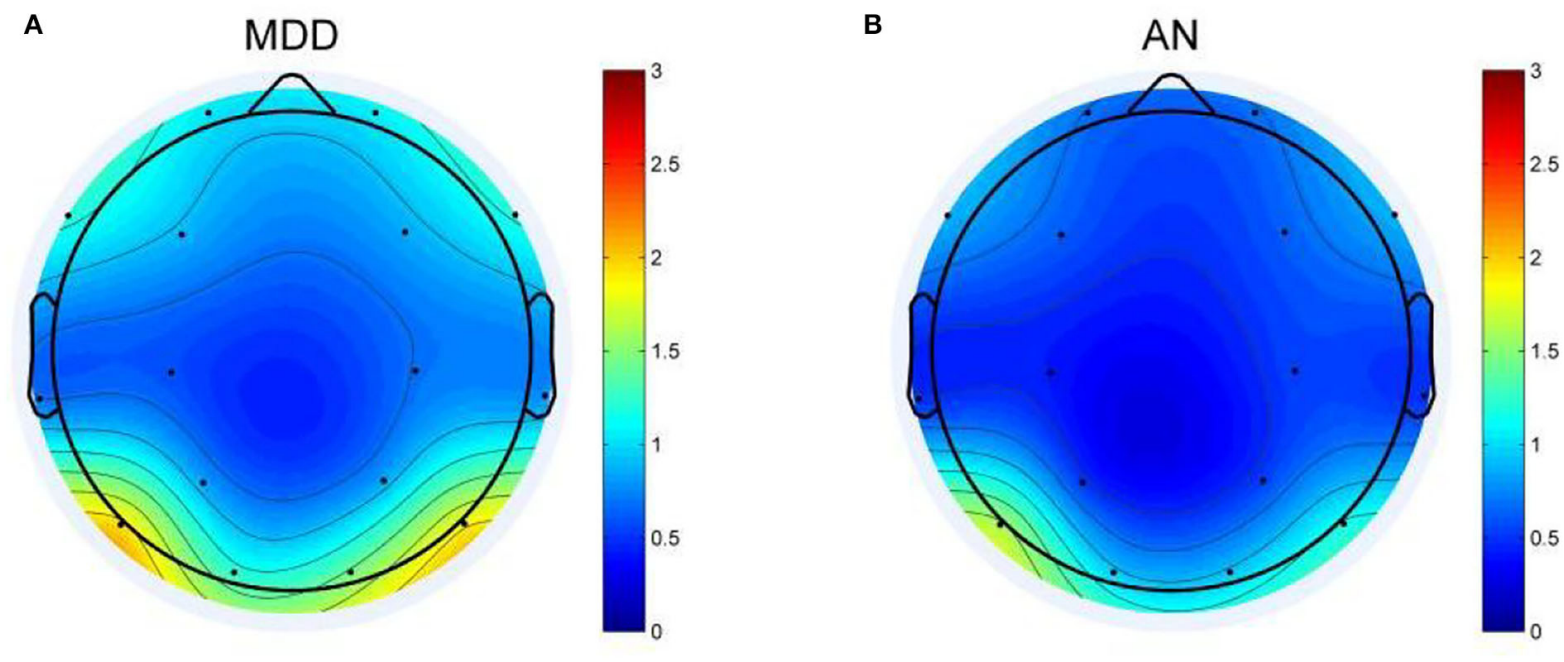
Topographic map of EEG power spectrum in the theta band for depression group (MDD) and anxiety group (AN). **(A)** The EEG power spectrum topography map of MDD group. **(B)** The EEG power spectrum topography map of AN group. The diagram above shows the difference in the energy distribution between the two groups. The blue part represents low EEG power, and the red part represents high EEG power. The darker the blue, the lower the power, and the darker the red, the higher the power.

**Figure 2:**
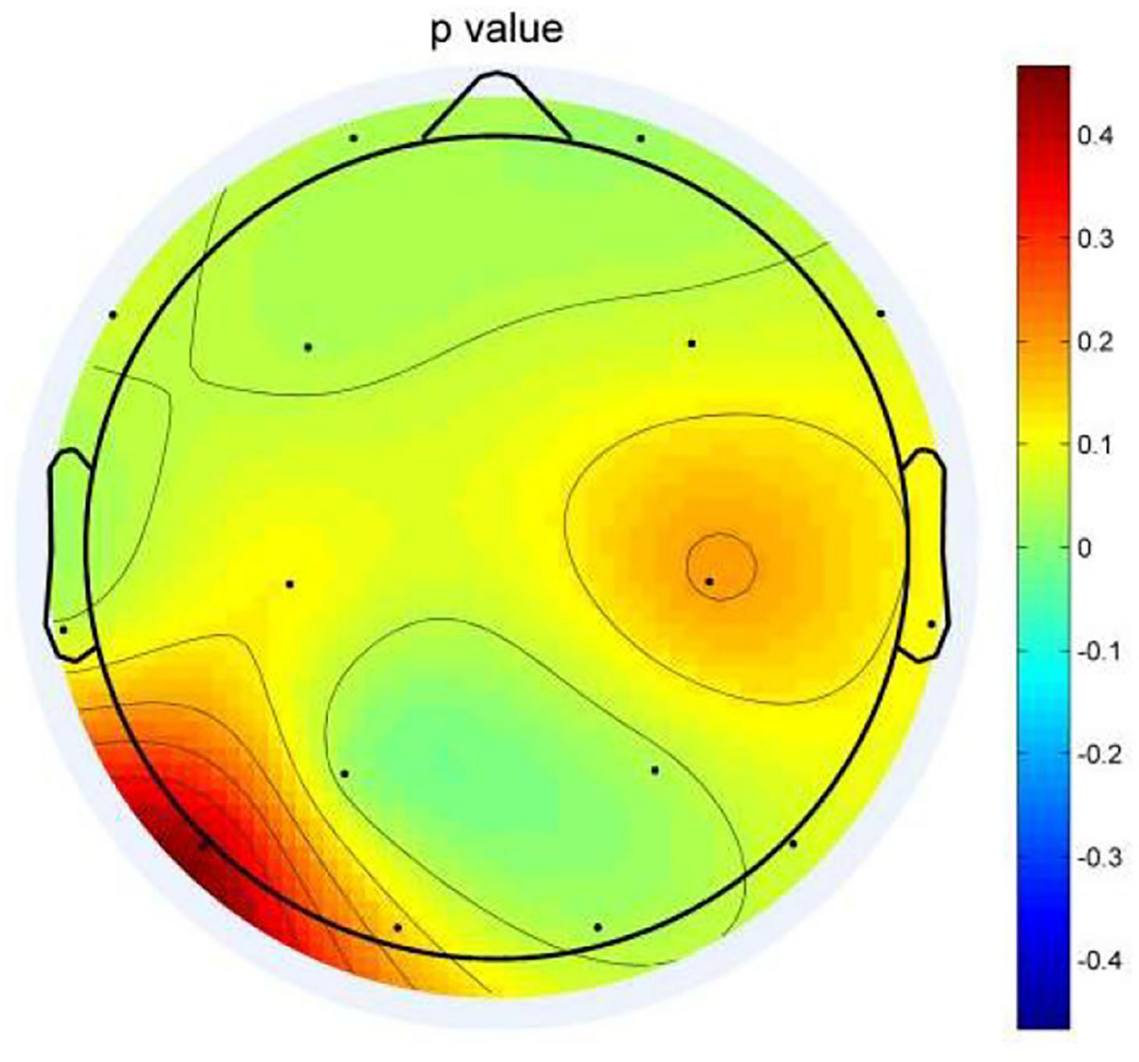
Topographic map of *P*-values between the two groups. Green areas represent significant differences (*P* < 0.05).

EEG analysis revealed differences between two groups in theta oscillations of 4–7 Hz at eight electrode sites (FP1, FP2, F3, O2, T3, P3, P4, and F8). Calculated using eight different homologous electrodes in theta power, these values are projected to cover the cerebral hemisphere. Power spectrum energy values are depicted in different colors on the topographic map. Accordingly, the theta band of 4–7 Hz can be plotted for the two groups (Figure 1). *P*-values of the region between the eight electrode positions indicate significance. To represent *p*-values between two groups, regions formed were divided by different colors. Figure 2 depicts the *p*-value of power spectral density between the two groups which can be utilized to obtain a more intuitive sense of their differences. Concerning the theta oscillations of 4–7 Hz, significant differences were found in green areas (P < 0.05).

The topographic map (Figure 2) illustrates differences in the regions of the prefrontal cortex (FP1, FP2, F3), left temporal lobe (T3), and right occipital regional (P3, P4, and O2). Among them, the difference is most obvious in the right occipital region (P3, P4, and O2). In the distributions in the left occipital cortex region (T5), little difference was observed between the two groups. The two groups exhibited a similar topographic distribution of the power spectrum of the two bands. Figure 1 depicts the EEG power spectrum distribution of anxiety patients, which is lower than depression patients. The two groups revealed higher value distribution in the posterior region, a lower value distribution in the central temporal region, and a slightly higher value distribution in the frontal region. Compared with depression patients, anxiety patients have asymmetrical distribution power of theta power on 4–7 Hz in the rear region. Significant differences between the two groups were more obvious on the left temporal lobe than on the right rear (Figure 2). Demographic differences between the two groups of patients were previously ruled out. Therefore, the above conclusion is logical.

**Figure 3:**
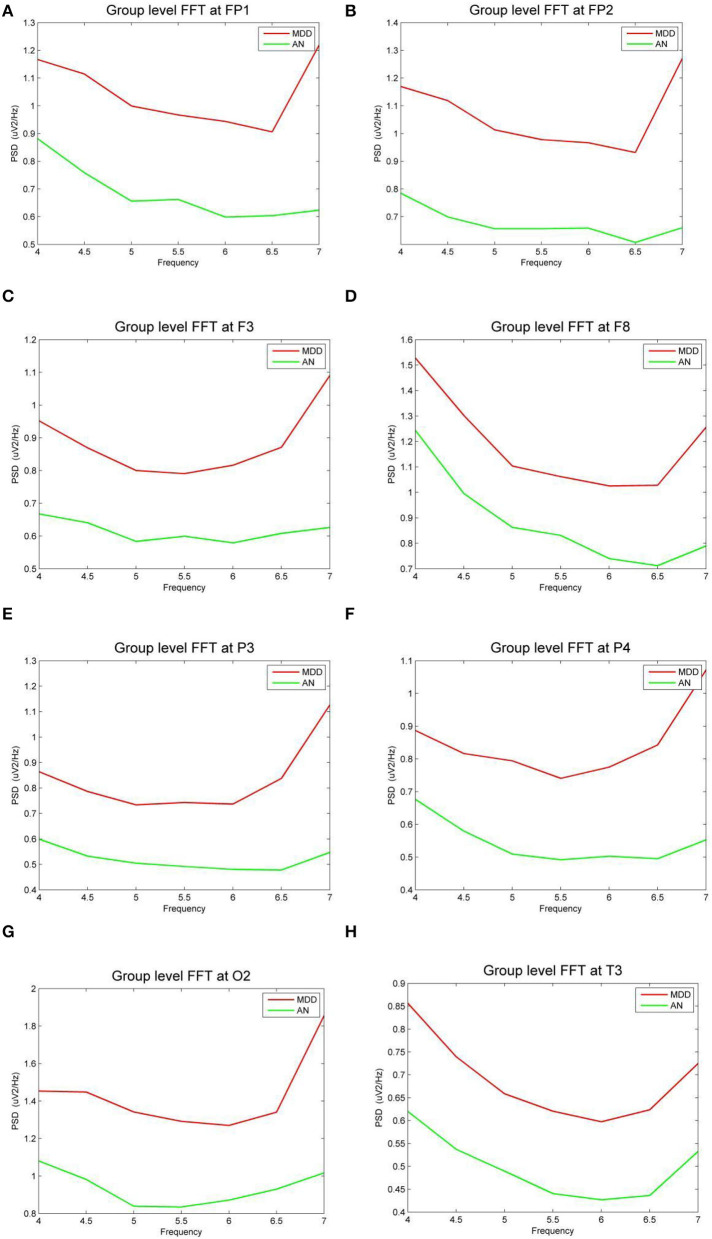
**(A–H)** The power spectral density (PSD) for the different electrode sites according depressive group (MDD) and anxiety group (AN). The horizontal axis represents the EEG frequency, the vertical axis represents the EEG PSD, the red line represents MDD group, and the green line represents AN group.

In eight bands, statistically significant differences in power spectral density were observed. Three electrodes in the prefrontal area (FP1, FP2, and F3) on theta frequency between two groups varies markedly (*P* < 0.05). The power of the depression group was obviously higher than the anxiety group (Figures 3A–C). The left temporal lobe, particularly at electrode T3, differed between the two groups (Figure 3H), whereas the right temporal lobe differences were found in the F8 electrode (Figure 3D). Differences were identified between the two groups in the temporal lobe. The power of the depression group revealed significantly stronger responses than the anxiety group, as evidenced by right occipital regional responses (Figures 3E–G). In the posterior occipital region, the energy difference between the two groups increased as the frequency increased and the difference between the two groups was most obvious at 7 Hz.

## Discussion

The aim of distinguishing MDD and AN is to better understand EEG biomarkers and facilitate the early diagnosis and effective treatment of both these diseases (31). EEG frequencies (alpha, beta, theta, and delta) are potential biomarkers of depression (32). MDD and AN differ in theta oscillations (4–7 Hz) and the results show that there are differences in the eight electrodes of F3, O2, T3, P3, P4, FP1, FP2, and F8. These eight different electrode points may represent the prefrontal cortex, left temporal, and right occipital regions.

### Differences in brain regions

Patients with mental illness experience altered brain activity. Patients with anxiety and those with depression showed different levels of activation and inhibition of brain activity in different brain regions. Hayata et al. (33) studied the difference in cerebral hemispheric activity in anxiety and depression using different brain activity pattern models of distinct regions. Bruder et al. (34) also came to a similar conclusion. As early as 1984, Robinson et al. (35) reported that damage to the left frontal lobe can lead to depression, and when the left brain is injured, the closer the lesion is to the frontal lobe, the more severe the depressive symptoms. Subsequent studies delved deeper into the link between prefrontal brain activity and depression. Hu et al. (36) found that MDD patients had significantly lower activation in the right prefrontal cortex. The right frontal lobe brain activity in persons with depression is reduced compared with the left side, and the approach motivation is weakened (37). Research by Mohamed Nour et al. (37) on the prefrontal cortex also suggests that changes in brain electrical activity may be closely related to emotional processing, social interaction, and cognition, and that low activation in this area may be associated with lower thresholds for sad and depressive experiences (38). Emotional behavior is associated with asymmetrical frontal lobe activation, negative emotion, or dragging behavior with right frontal lobe activation, and positive emotion or approach behavior with left frontal lobe activation (38). The different changes in electrical activity in the bilateral prefrontal cortex caused by anxiety and depression may explain the differences in the EEG power of the prefrontal cortex between the two groups of patients in the results of this study.

Previous neuroimaging studies have shown that the temporal lobe can act as a network node in anxiety disorders (39–43). Anxiety leads to redistribution of regional cerebral blood flow (rCBF) (31), which decreases relative to left frontal EEG activity reflecting decreased approach motivation or increased withdrawal tendencies (44). Additionally, activation of marked brain activity was decreased in the right temporal lobe in patients with major depressive disorder (45). The difference in the temporal lobe EEG power in this study is related to the functional loss caused by the decreased left hemisphere cerebral blood flow in anxiety patients and the weakened right temporal lobe electrical activity in depressed patients. Asymmetries of brain activity in different brain regions (especially in the region of prefrontal cortex and left temporal lobe) in patients with depression and anxiety are associated with specific symptom characteristics of the disease.

There are few previous studies on depression and anxiety disorders in the occipital area, and only a few research suggest that the generation of anxiety may be related to the changes in the brain electrical activity in the occipital area (40). The connection between mental illness and the occipital area needs to be further verified.

### Study in theta oscillations

Theta rhythm can be a good indicator in diagnostic tools (46–56). As two major psychiatric illnesses, anxiety is inextricably linked to depression (57). This study revealed that almost 50% of adults with a 12-month history of GAD met the criteria for lifelong major depressive disorder, compared to only 7.4% of those without GAD (58). Theta bands associated with emotional processing (48) are well relative to depression and healthy controls (59). Studies indicate that a higher baseline theta activity is associated with greater improvement in depression (46, 47). Additionally, frontal theta asymmetry is also a potential biomarker of depression (17, 32). In 2000, Suetsugi et al. (9) suggested that frontal midline theta is a reliable measure of anxiety and that low levels of theta are associated with higher levels of anxiety. In other words, the theta power distribution at the group level was negatively correlated with the severity of anxiety attacks. Therefore, theta oscillations may be a biomarker for differentiating depression and anxiety disorders.

As one of the biomarkers of EEG, the relationship between theta oscillations and mental disorders that affect brain activity is still worthy of further study. Previous research has indicated a variation in EEG activity on theta frequency in patients with depression (60). Depression has been demonstrated to increase theta band activity in occipital and parietal regions (61), which can reflect a decreased cortical activation in these brain regions. Meanwhile, theta oscillations may be associated with negative clinical symptoms in patients with anxiety and depression, such as seeking multiple ways to engage in suicidal or self-harming behaviors (62, 63). Theta relative power in the central frontal region (F3, FZ, FCZ, and CZ) was significantly higher in the group with high Scale for Suicidal Ideation (SSI) scores than with low SSI scores (64). Obviously, the severity of depressive symptoms is proportional to theta power. In the theta band, anxiety increased connectivity between the right frontal and central areas and right temporal and left occipital areas (15). Theta rhythm may be closely related to the electrical activity in the medial prefrontal cortex (65), which has been linked to various neurological and psychiatric disorders, including MDD and AN (66).

In conclusion, theta oscillation might be of some value in distinguishing MDD and AN; the prefrontal lobe, left temporal lobe, and right occipital lobe are significantly different in patients with anxiety and those with depression. However, further studies are needed to confirm the importance of the EEG biomarker theta oscillation in psychiatric disorders.

## Limitations

Certain limitations of the results should be considered. On the one hand, in terms of data, the sample size was limited and the number of EEG leads were insufficient. The small sample size may have had limitations in terms of experimental age and gender. The EEG data that were collected was from a single lead, affecting the experimental results. At this stage, some of the more advanced EEG research leads have reached 256 leads. The increase in the number of EEG leads can increase the accuracy of brain region localization and is also critical for further exploration of diseases. At the same time, the influence of the style of the electrode cap and the material of the EEG paste on the research results cannot be ignored. On the other hand, most clinical diagnostic tools rely on medical history collection and self-reporting. Although medical history and scales have been used as the basis for clinical diagnosis for many years, there are still some deficiencies, such as insufficient sensitivity and specificity. Finally, this study is limited to the generalized level of psychiatric disorders and did not conduct research at the subtype level.

To address the shortcomings of existing research, future research should first expand the sample size, choose EEG caps with more leads, test experimental equipment multiple times, such as EEG caps of different materials and EEG heights, and then select EEG data. Future research can also explore the relationship between disease subtypes, such as the difference between depression with or without anxiety symptoms. The relationship between EEG biomarkers and psychiatric disorders other than EEG biomarker theta oscillations, which was mainly explored in this study, can also be explored.

## Conclusion

Compared to the depression group, the anxiety group demonstrated a decrease in power. The differences between the two groups are concentrated in the prefrontal cortex, left temporal lobe, and right occipital regions, affecting emotional regulation and cognitive functions. Theta frequencies enable early identification of depressive and anxious symptoms, as well as effective and objective treatment monitoring. Additional research is warranted to explore whether other EEG biomarkers would be beneficial in clinical studies.

## Data availability statement

The raw data supporting the conclusions of this article will be made available by the authors, without undue reservation.

## Ethics statement

The studies involving human participants were reviewed and approved by the First Hospital Committee of Shanxi Medical University. The patients/participants provided their written informed consent to participate in this study.

## Author contributions

YZ: study design, data curation, formal analysis, and writing—original draft. LL: investigation, validation, and writing—review and editing. ZiL and MG: investigation, software, and writing—review and editing. ZhL and NS: investigation, resources, and writing—review and editing. CY and AZ: carried out recruitment, clinical treatment, and writing—review and editing. YW: investigation and writing—review and editing. KZ: funding acquisition, advisors, overall oversight, and writing—review and editing. All authors contributed to the article and approved the submitted version.

## Funding

This study was supported by the National Key Research and Development Program of China (2016YFC1307103), the National Natural Science Foundation of China (No. 81471379), the National Natural Science Foundation of China (81701345), and the Natural Science Foundation of Shanxi Province for Youths (201601D021151).

## Conflict of interest

The authors declare that the research was conducted in the absence of any commercial or financial relationships that could be construed as a potential conflict of interest.

## Publisher's note

All claims expressed in this article are solely those of the authors and do not necessarily represent those of their affiliated organizations, or those of the publisher, the editors and the reviewers. Any product that may be evaluated in this article, or claim that may be made by its manufacturer, is not guaranteed or endorsed by the publisher.
